# Fentanyl conjugate vaccine by injected or mucosal delivery with dmLT or LTA1 adjuvants implicates IgA in protection from drug challenge

**DOI:** 10.1038/s41541-021-00329-0

**Published:** 2021-05-13

**Authors:** Addison E. Stone, Sarah E. Scheuermann, Colin N. Haile, Gregory D. Cuny, Marcela Lopez Velasquez, Joshua P. Linhuber, Anantha L. Duddupudi, Jennifer R. Vigliaturo, Marco Pravetoni, Therese A. Kosten, Thomas R. Kosten, Elizabeth B. Norton

**Affiliations:** 1 Department of Microbiology and Immunology, Tulane University School of Medicine, New Orleans, LA USA; 2 Department of Psychology, University of Houston, Houston, TX USA; 3 Texas Institute of Measurement Evaluation and Statistics, University of Houston, Houston, TX USA; 4 Department of Pharmacological & Pharmaceutical Sciences, University of Houston, Houston, TX USA; 5 Department of Pharmacology, University of Minnesota Medical School, Minneapolis, MN USA; 6 Center for Immunology, University of Minnesota, Minneapolis, MN USA

**Keywords:** Adjuvants, Conjugate vaccines

## Abstract

Fentanyl is a major contributor to the devastating increase in overdose deaths from substance use disorders (SUD). A vaccine targeting fentanyl could be a powerful immunotherapeutic. Here, we evaluated adjuvant and delivery strategies for conjugate antigen vaccination with fentanyl-based haptens. We tested adjuvants derived from the heat-labile toxin of *E. coli* including dmLT and LTA1 by intramuscular, sublingual or intranasal delivery. Our results show anti-fentanyl serum antibodies and antibody secreting cells in the bone-marrow after vaccination with highest levels observed with an adjuvant (alum, dmLT, or LTA1). Vaccine adjuvanted with LTA1 or dmLT elicited the highest levels of anti-fentanyl antibodies, whereas alum achieved highest levels against the carrier protein. Vaccination with sublingual dmLT or intranasal LTA1 provided the most robust blockade of fentanyl-induced analgesia and CNS penetration correlating strongly to anti-FEN IgA. In conclusion, this study demonstrates dmLT or LTA1 adjuvant as well as mucosal delivery may be attractive strategies for improving the efficacy of vaccines against SUD.

## Introduction

The United States is in the midst of a nationwide opioid crisis, with an estimated 2.1 million individuals suffering from an opioid use disorder (OUD)^1^. Particularly alarming are the increasing fatal overdoses resulting from illicit fentanyl (FEN). Higher incidence of drug overdose deaths was proportionally associated with the number of FEN seizures in a given community^2^. FEN is believed to specifically account for nearly 30,000 of the 50,000 deaths involving opioids annually, due to its high potency, ease of manufacturing, and/or addition to other illicit drugs unknowing to the user^3–5^. Since the 2020 COVID-19 pandemic, there is new evidence that FEN use and opioid overdoses may also be increasing^6^. FEN and FEN-analogs rapidly penetrate the central nervous system and are 50–100 times more potent than heroin and morphine respectively. Because of this potency as well as its half-life, higher or repeated doses of the mu opioid receptor antagonist naloxone (Narcan^©^) are needed to be administered quickly to reverse lethal overdoses^7^.

One option to help combat this opioid epidemic is vaccination. A vaccine specifically targeting illicit opioid(s) could attenuate the reinforcing effects of FEN and prevent overdose deaths by preventing the drug from penetrating the CNS, particularly in individuals recovering from OUD during critical periods when relapse is especially common. Such a vaccination strategy would induce serum antibodies that diminish drug activity by assembling an antibody-drug complex that is too large to cross the blood–brain barrier, but not prevent other pain medications (e.g., tramadol, morphine) from working. Vaccines for substance use disorders (SUDs) have been under development for decades, including for cocaine, nicotine, heroin, amphetamines, and other synthetic opioids but without commercial success and FDA approval^8–13^. As observed with cocaine and nicotine vaccines in Phase III clinical trials, a lack of sustainable antibody levels and consistent levels of antibodies in vaccinated subjects has been a persistent problem^14–18^. Yet subjects who attained high levels of antibodies against nicotine or cocaine following vaccination showed significantly reduced drug use compared to those that only achieved low levels of antibodies and the placebo group^14,19^.

FEN is not naturally immunogenic, so the drug must be conjugated to a protein in order to create an immunogenic vaccine antigen. Recent animal studies with various opioid conjugate immunogens have demonstrated development of antibodies to FEN or heroin resulting in decreasing potency of heroin and FEN distribution to the brain after experimental exposure, reducing the drug-induced antinociception (or blocking of pain)^9–13,20–24^. However, a frequent problem of these conjugate vaccines is still short duration of immunity and/or insufficient antibody magnitudes even with an added adjuvant. As recently reviewed^25^, no single adjuvant has emerged as ideal for vaccines against SUDs, though alum, TLR-based agonist, or combinations thereof have been tested. Alum, or aluminum salts, are the most commonly used adjuvants in licensed vaccines; evaluations of alum in drug abuse or addiction vaccines thus far has had limited success in inducing an enduring antibody response^13,25–27^. Few studies have shown efficacy for routes other than injected delivery (e.g., by intramuscular (IM), subcutaneous, and intraperitoneal routes), with a few exceptions in vaccines for cocaine^28^. Mucosal vaccination has an advantage of needle-free delivery, lack of injection-related infections, possibility of non-medical personnel administration or self-administration, and induction of robust systemic and mucosal immunity^29–31^. In addition, mucosal vaccination can alter the quality of antibody isotypes, particularly IgA, and may better protect against mucosal drug exposures, (e.g., snorting or smoking), than parenteral vaccination.

A newer class of adjuvants being developed are derived from heat-labile enterotoxins from *E. coli* (LT) or *V. cholerae*. These proteins have an enzymatic A-subunit critical for adjuvant effects^32–35^ that ADP-ribosylates Gsα, leading to irreversible adenylate cyclase activation and accumulation of intracellular cAMP. dmLT or LT(R192G/L211A) is the most advanced and clinically relevant protein from this adjuvant family^36^. dmLT improves parenteral and mucosal immunity to bacterial and viral antigens following a variety of routes in animal models, including IM and sublingual (SL) delivery routes^37–43^ and has been tested in a number of recent human clinical trials^44–46^. In addition, dmLT enhanced antibody responses to conjugate antigens using a polysaccharide-protein conjugate against *V. cholerae*^47^. We have also developed a B-subunit free adjuvant called LTA1 based on the A1 domain^39,48,49^. LTA1 was specifically developed for safe intranasal (IN) use to overcome the risk of Bell’s palsy with AB_5_ LT proteins^48,50,51^. Like dmLT, LTA1 also activates antigen presenting cells^49^ and stimulates immunity to parenteral injection of vaccine antigen^52^. However, the ability of dmLT, LTA1, or any bacterial-enterotoxin based adjuvant for vaccines targeting substances associated with use disorders has not been explored (though the cholera toxin binding B-subunit has been used as a carrier for cocaine vaccines^16^).

The objective of this study was to test whether LT-based adjuvants can enhance the efficacy of a candidate FEN vaccine. To test this, we evaluated two FEN conjugate immunogens in combination with dmLT, LTA1, or alum adjuvants and administered to mice by IM, SL, or IN routes.

## Results

### IM vaccination with FEN-BSA conjugate admixed with dmLT results in robust FEN-specific serum IgG and antibody secreting cells (ASCs) in the spleen and bone marrow

**Fig. 1:**
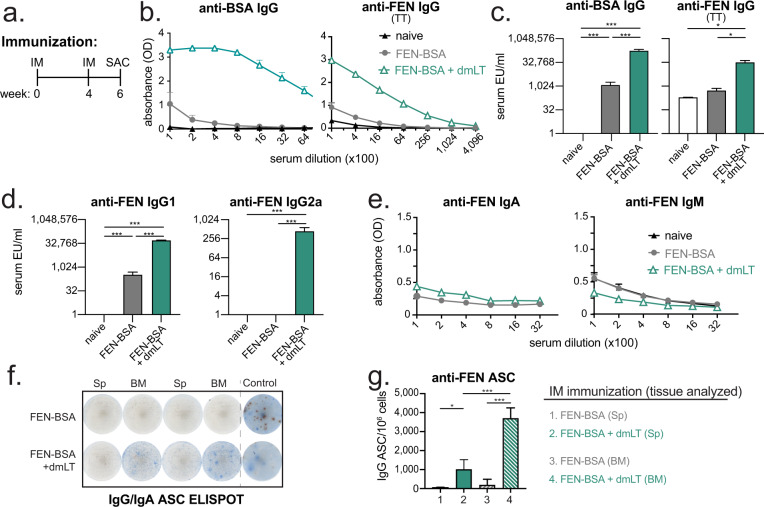
Immune responses to intramuscular immunization with FEN-BSA are enhanced with dmLT adjuvant. Groups of Balb/c mice (*n* = 4) were left naïve or immunized intramuscularly with 8 μg FEN-BSA ± 1 μg dmLT on weeks 0 and 4. Two-weeks later (week 6) serum, spleen, or bone-marrow were collected for antibody evaluations. **a** Schematic of immunization schedule identifying week of immunizations (IM) and sacrifice for blood, spleen, and bone-marrow sample collections (SAC). **b** Raw ELISA data for serum anti-BSA or anti-FEN IgG expressed as 405 nm optical density (OD) absorbance. For anti-FEN IgG, plates were coated with FEN-TT. **c** Compiled ELISA units (EU)/ml of indicated serum anti-BSA and anti-FEN IgG titers assessed by ELISAs graphed on a log2 scale. **d** Compiled EU/ml of indicated serum anti-FEN IgG1 or IgG2a. **e** Raw ELISA data for serum anti-FEN IgA and IgM. **f** Representative images for ASCs IgG/IgA ELISPOT results using splenocytes (Sp) or bone marrow (BM) cells and plates coated with FEN-TT. Blue spots indicate IgG ASCs and red spots IgA ASCs. **g** Compiled ASC per 10^6^ cells from spleens or bone marrow. Bars at mean + s.e.m. with significance indicated as (***) for *P* ≤ 0.001 (*) for *P* ≤ 0.05 by one-way ANOVA with Bonferroni’s multiple comparisons test.

We first vaccinated mice with 8 μg of a commercially available FEN-BSA conjugate antigen alone or admixed with 1 μg dmLT. These formulations were delivered by prime/boost IM immunization to mice (week 0, 4) with sample analyses performed two weeks after the second immunization (week 6, Fig. 1a) compared to naïve mice. We observed a statistically significant increase in serum antibodies to carrier antigen, using ELISA plates coated with BSA, as well as specific to FEN, using ELISA plates coated with FEN-TT, in all vaccinated mice compared to naïve mice; however, this was highest when dmLT was included in the vaccine formulation (Fig. 1b–c, F-statistics and degrees of freedom reported in Supplemental Table 1). The majority of induced anti-FEN antibodies were an IgG1 isotype, with some IgG2a, but not IgA or IgM (Fig. 1d–e). To test if serum antibodies corresponded with an increased number of antigen-specific ASCs, bone-marrow and spleen tissues were also analyzed for anti-FEN ASCs by ELISPOT. We found a statistically significant increase in the number of anti-FEN IgG ASCs in FEN-BSA + dmLT vaccinated mice compared with FEN-BSA vaccinated mice in either tissue (Fig. 1f–g). This was maximal in bone-marrow, where memory B-cells and long-lived plasma cells typically reside.

### Anti-FEN immunity following prime/boost IM vaccination with FEN-CRM_197_ conjugate admixed dmLT or LTA1 is superior to alum or antigen alone

**Fig. 2:**
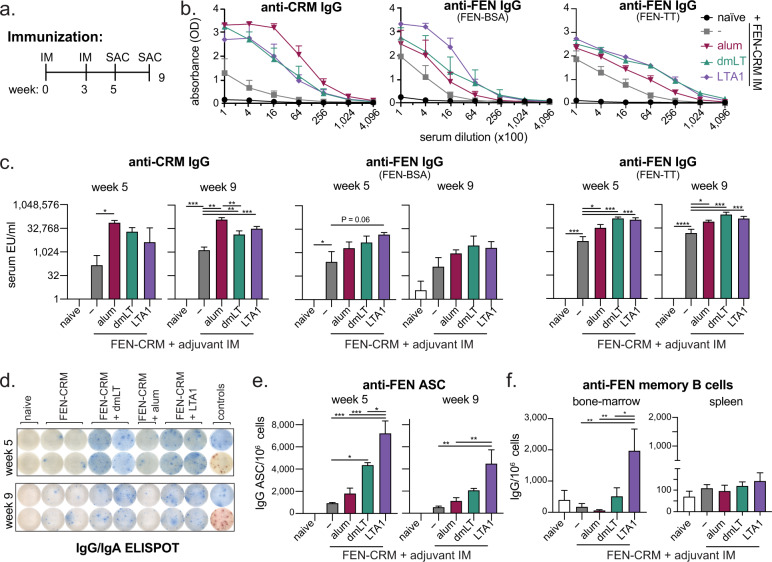
Immune responses to prime/boost intramuscular immunization with FEN-CRM_197_ are enhanced in magnitude and longevity with dmLT and LTA1 adjuvants. Groups of Balb/c mice (*n* = 3–5) were left naïve or immunized IM with 5 μg FEN-CRM ± 150 μg alum, 0.1 μg dmLT, or 5 μg LTA1 on weeks 0 and 3. Two-weeks later (week 5) or six-weeks later (week 9) serum, spleen, and bone-marrow was collected for antibody evaluations. **a** Schematic of the immunization schedule. **b** Raw ELISA data for week 5 serum anti-CRM or anti-FEN serum IgG expressed as absorbance. Two anti-FEN ELISA coating antigens were used as indicated by FEN-BSA or FEN-TT. **c** Compiled week 5 and 9 serum anti-CRM or anti-FEN (coating antigen indicated) serum IgG. **d** Representative anti-FEN (FEN-TT coating antigen) IgG/IgA ELISPOT images from bone-marrow of week 5 immunized mice. Blue spots indicate IgG and red spots IgA ASCs. **e** Compiled anti-FEN IgG ASC per 1e6 cells from bone marrow. **f** Anti-FEN IgG memory B cells per 10^6^ cells differentiated in culture from immunized splenocytes from week 9 post immunization. Bars at mean + s.e.m. with significance determined by ANOVA paired with Bonferroni post-hoc test as shown (*P < 0.05, **P < 0.01, ***P < 0.001).

Based on promising results using the FEN-BSA antigen above and published FEN hapten conjugation strategies^13^, we prepared a FEN-CRM_197_ (FEN-CRM) antigen (Supplemental Fig. 1). The *E. coli*-expressed CRM_197_ protein, or EcoCRM, is a low-cost manufactured protein derived from detoxified diphtheria toxin^53^ with fewer complications reported from pre-existing immunity compared to other common carrier proteins^54,55^. It has successfully been used in a recent pre-clinical heroin vaccine study^26^. To test the efficacy of the FEN-CRM antigen as well as adjuvant combinations in generation of anti-FEN immunity, we next immunized groups of mice with 5 μg FEN-CRM alone or admixed with 0.1 μg dmLT, 5 μg LTA1, or absorbed 1:1 with 2% alhydrogel (alum). Since responses to substance abuse vaccines have not been optimal with alum^13^, we aimed to have higher responses with dmLT or LTA1 adjuvanted vaccination than observed with the alum group. Formulations were delivered by prime/boost IM to mice (weeks 0 and 3) followed by immunologic analyses at two-weeks (week 5) or 6 weeks later (week 9, Fig. 2a). We observed a statistically significant increase in serum antibodies to CRM_197_ carrier antigen in all adjuvanted groups compared to antigen-alone vaccination, which was highest in the alum adjuvanted group (Fig. 2a–b). In contrast, antibody responses specific to FEN, determined using ELISA plates coated with FEN-BSA or FEN-TT, were highest in dmLT and LTA1 adjuvanted groups. These observed serum antibody responses were evident even 6 weeks after the boost (week 9). As before, we also evaluated vaccinated animals for number of anti-FEN ASCs in the bone-marrow (tissue selected based on Fig. 1 experiments). We observed ASCs in all groups that were vaccinated with FEN-CRM antigen, with significantly higher levels in dmLT and LTA1 adjuvanted groups compared with antigen alone, but not in the alum adjuvanted group (Fig. 2d–e). Evaluation of memory B cells in bone-marrow or spleen, using 5 days of ex vivo expansion and differentiation into ASC with mitogens using standard methods^56^, revealed only the LTA1 adjuvanted group in bone-marrow tissue exhibited significant memory B cells after vaccination compared to all other FEN-CRM groups (Fig. 2f). Similar findings were also observed with a shorter time between vaccination boosters and a smaller dose of antigen (Supplemental Fig. 2).

### Anti-FEN immunity following three IM vaccinations with FEN-CRM197 conjugate admixed dmLT or LTA1 is superior to alum or antigen alone

**Fig. 3:**
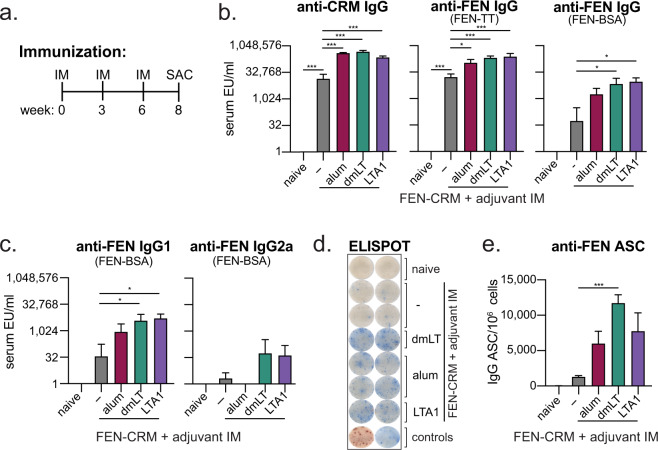
Immune responses to prime/boost/boost intramuscular FEN-CRM197 immunization are enhanced in magnitude with dmLT and LTA1 adjuvants. Groups of Balb/c mice (*n* = 3–5) were left naïve or immunized IM with 5 μg FEN-CRM ± 150 μg alum, 0.1 μg dmLT, or 5 μg LTA1 on weeks 0, 3, and 6. Two-weeks later (week 8) serum and bone-marrow was collected for antibody evaluations. **a** Schematic of immunization schedule. **b** Compiled Week 5 and Week 9 serum anti-CRM or anti-FEN (coating antigen indicated) serum IgG. **c** Compiled EU/ml of indicated serum anti-FEN serum IgG1, or IgG2a ELISAs. **d** Representative anti-FEN (TT-BSA coated) IgG/IgA ELISPOT images from bone-marrow of week 8 immunized mice. Blue spots indicate IgG and red spots IgA ASCs. **e** Compiled anti-FEN IgG ASC per 10^6^ cells from bone-marrow. Bars at mean + s.e.m. with significance determined by ANOVA paired with Bonferroni post-hoc test as shown (*P < 0.05, **P < 0.01, ***P < 0.001).

We observed higher levels of anti-FEN immunity with dmLT and LTA1 adjuvants and FEN-CRM after prime/boost immunization (Figs. 1–2). To test how an additional booster immunization would change the magnitude of immunity post vaccination, we repeated our immunizations from above (Fig. 2) except on a prime/boost/boost schedule on weeks 0, 3, and 6, with sample collection on week 8 (Fig. 3a). As before, FEN-CRM alone or adjuvanted with alum, dmLT or LTA1 resulted in serum antibodies and ASCs to CRM carrier or FEN (Fig. 3b–e). Immune responses to carrier were more similar between adjuvant groups (than with two immunizations in Fig. 2), but higher levels of anti-FEN serum IgG, IgG1, or IgG2a or anti-FEN IgG ASCs were observed in dmLT and LTA1 groups. No clear differences between dmLT and LTA1 groups were observed in serum antibody analyses; however, the dmLT group had the higher number of IgG ASCs when compared to FEN-CRM vaccination alone (Fig. 3e).

### Anti-FEN immunity is promoted with mucosal booster immunizations, including dmLT SL or LTA1 IN vaccination

**Fig. 4:**
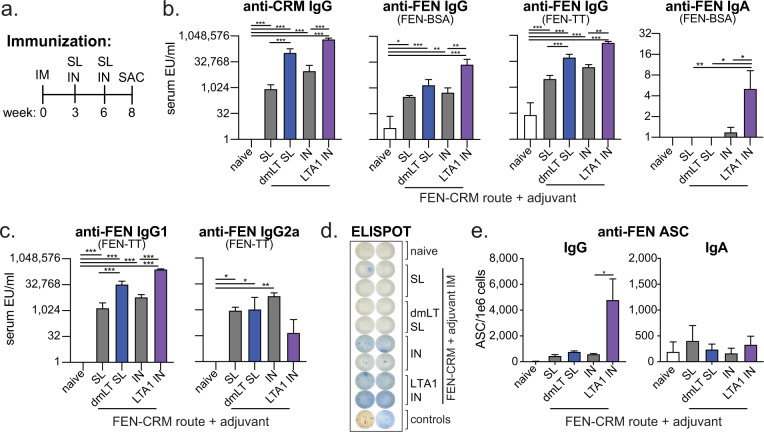
Immune responses to FEN-CRM_197_ can be generated by sublingual or intranasal booster immunizations containing either dmLT or LTA1 adjuvants. Groups of Balb/c mice (*n* = 6) were left naïve or primed by 5 μg FEN-CRM ± 0.1 μg of dmLT or 5 μg of LTA1 by IM delivery. Mice were boosted mucosally on weeks 3 and 6 with 9–10 μg FEN-CRM ± 5 μg dmLT SL or 9 μg FEN-CRM ± 5 μg LTA1 IN, both in 30 μl. On week 8, serum and bone-marrow were collected for immune response evaluation. **a** Schematic of immunization schedule. **b** Compiled serum anti-CRM and anti-FEN (FEN-BSA, FEN-TT) serum IgG ELISAs. **c** Compiled serum anti-FEN (FEN-TT) serum IgG1 or IgG2a ELISAs. **d** Representative anti-FEN (TT-BSA coated) IgG/IgA ELISPOT images from bone-marrow. Blue spots indicate IgG and red spots IgA ASCs. **d** Compiled serum anti-FEN (FEN-BSA) IgA ELISA. **e** Compiled anti-FEN IgG ASC per 10^6^ cells from bone-marrow. Bars at mean + s.e.m. with significance determined by ANOVA paired with Bonferroni post-hoc test as shown (*P < 0.05, **P < 0.01, ***P < 0.001).

While our studies thus far utilized IM delivery, both dmLT and LTA1 are unique adjuvants because they are also highly effective by mucosal delivery. Past animal and human studies have shown that immunization by sublingual (SL) delivery with dmLT plus antigen directly to the mucosal tissue under the tongue^37,43,57–62^ or animal studies with immunization by intranasal (IN) delivery with LTA1 + antigen to the nasal epithelium^39,48^ are safe and promote vaccine immunity. No mucosal vaccinations have previously been reported with OUD vaccines, but presumably could be advantageous for outpatient or self-delivery immunization schemes, or to counteract intranasal (snorting) or intrapulmonary (smoking) delivery of opioids. To test whether mucosal delivery alone or with adjuvant could be effective as booster immunizations, we performed additional animal vaccinations selecting antigen/adjuvant doses based on previous studies^48,52^. Mice were first primed by IM delivery as before (e.g., Figs. 2–3) with 5 μg FEN-CRM alone or admixed with 0.1 μg dmLT or 5 μg of LTA1. This was followed by booster vaccinations with 9–10 μg FEN-CRM alone or admixed with 5 μg dmLT by SL delivery or 5 μg LTA1 by IN delivery on weeks 3 and 6. Sample collection was performed on week 8 (Fig. 4a). FEN-CRM immunization with mucosal boosting with and without dmLT or LTA1 adjuvants resulted in serum antibodies and ASCs to CRM carrier and/or FEN (Fig. 4b–e). Higher levels of anti-FEN serum IgG, IgG1, and anti-FEN IgG ASCs were observed in dmLT SL and LTA1 IN groups, with highest levels in the latter. LTA1 IN group also had significant levels of anti-FEN serum IgA, but less IgG2a than the IN (no adjuvant) group (Fig. 4c). Significant levels of IgA ASCs in the bone-marrow were not observed (Fig. 4e). Similar findings were also observed with SL booster vaccination with FEN-BSA with and without dmLT, with serum IgA also being detected in this experiment (Supplemental Fig. 3).

### Anti-FEN serum antibodies or ASCs are significantly correlated for IgG but not for IgA

**Fig. 5:**
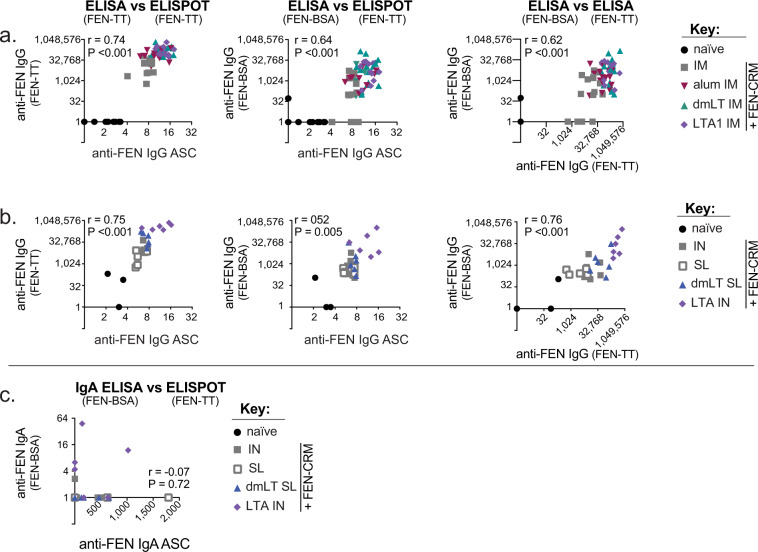
Significant correlations between anti-FEN IgG antibody analyses by serum ELISAs and ASC ELISPOT. **a** Correlations between indicated IgG ELISAs or ELISPOT results with coating antigen indicated in parenthesis using compiled data from FEN-CRM ± adjuvants IM prime/boost or prime/boost/boost experiments. **b** Correlations between indicated IgG ELISAs or ELISPOT results with coating antigen indicated in parenthesis using compiled data from FEN-CRM ± adjuvants prime IM and mucosal (SL or IN) booster experiments. **c** Correlations between indicated IgA ELISAs or ELISPOT results with coating antigen indicated in parenthesis using compiled data from FEN-CRM ± adjuvants prime IM and mucosal (SL or IN) booster experiments. ELISPOT data graphed as bone marrow ASC per 1e6 cells (log10). ELISA data graphed as serum IgG EU/ml (log2). Spearman correlations *P* values and correlation coefficient (*r*) indicated on each graph.

**I**mmunologic analyses of FEN conjugate vaccinations require a different FEN hapten conjugate to evaluate the immune responses. Thus far, we had observed slight differences in vaccination outcomes depending upon specific assay or coating antigen, with higher levels of antibodies when FEN-TT coating antigen was used (Figs. 1–3). To better compare the results of these assays, data were compiled and compared from IM experiments (Fig. 5a) or from IM prime with mucosal boosts (Fig. 5b, c). We observed that all anti-FEN IgG comparisons (e.g., FEN-BSA ELISA vs FEN-TT ELISA vs FEN-TT ELISPOT) were significantly and positively correlated using Spearman’s correlation test (*r* values between 0.52 and 0.76). However, limit of detection, background (estimated from naïve groups), and overall magnitude of observed responses were assay specific. These data indicate that the selection of a FEN-hapten conjugate for ELISA analyses can impact quantification of anti-FEN serum IgG antibodies. For anti-FEN IgA comparisons (e.g., FEN-BSA ELISA vs FEN-TT ELISPOT) serum antibodies and bone-marrow ASCs were not significantly correlated (Fig. 5c), indicating that another tissue area likely related to mucosal vaccination is serving as the niche for IgA ASCs. Importantly, these comparisons indicate that serum IgG antibodies are likely being produced by the ASCs found in the bone-marrow, cells critical for maintenance of antibodies in systemic circulation; however, circulating IgA antibodies are likely being produced by ASCs in mucosal tissue or draining secondary lymphoid organs yet to be identified.

### Protection from FEN-induced antinociception and brain tissue distribution after FEN-CRM immunizations is observed with parenteral and mucosal vaccination with dmLT and LTA1 adjuvants, but not with alum

**Fig. 6:**
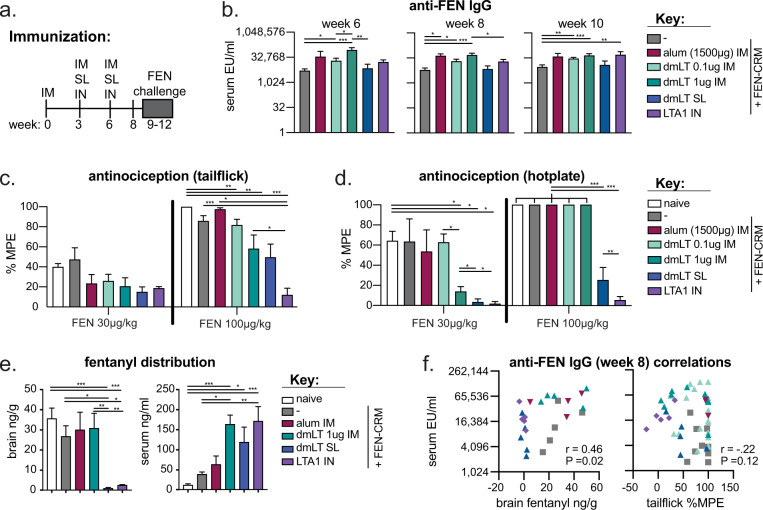
Reduction in fentanyl-induced antinociception and brain tissue distribution in dmLT and LTA1 vaccinated animals compared to alum or antigen alone groups. Groups of Balb/c mice (*n* = 5–20) were left naïve or immunized on weeks 0, 3, and 6 with FEN-CRM IM or with adjuvant and route combinations as performed previously. These included 1500 μg alum IM, 0.1–1 μg dmLT IM, 0.1 μg dmLT IM + 5 μg dmLT SL boosters (dmLT SL), or 5 μg LTA1 IM + 5 μg LTA1 IN boosters (LTA1 IN). FEN-CRM was administered at 5 μg for IM delivery and 9–10 μg for mucosal delivery. Blood was collected on week 6, 8, and 10 for serum analyses and mice challenged with fentanyl doses during weeks 9–12. Nociception tests were conducted using tail flick and hotplate assays with tissue taken for drug quantification. **a** Schematic of immunization schedule. **b** Compiled serum anti-FEN (FEN-BSA) serum IgG ELISA from week 6, 8, and 10 tailbleeds. **c** % maximum possible effect (MPE) from tail flick antinociception evaluation. **d** % MPE from hotplate antinociception evaluation. **e** Fentanyl concentrations in brain and serum after a final 0.1 mg/kg fentanyl challenge. **F** Correlation between brain fentanyl levels or tail flick antinociception %MPE vs anti-FEN serum IgG for immunized groups. Bars at mean + s.e.m. with significance determined by ANOVA paired with Bonferroni post-hoc test as shown (*P < 0.05, **P < 0.01, ***P < 0.001). Spearman correlations *P* values and correlation coefficient (*r*) indicated on relevant graph.

Next, we evaluated the protective effects of adjuvanted vaccination against FEN challenge. To do this we repeated select vaccination groups, including 5 μg of FEN-CRM alone, or with 1500 μg alum, 0.1 or 1 μg dmLT by IM prime/boost/boost. This higher dose of alum was chosen to improve upon the anti-FEN antibody responses observed with the 150 μg alum used in Figs. 2–4. This required a formulation volume of 100 μl for the 1500 μg alum group (delivered by 50 μl injection in both hind limbs), whereas all other IM injections were delivered at 20 μl as used previously. We also included mucosal booster groups (as in Fig. 4) including dmLT IM+SL+SL and LTA1 IM+IN+IN. All mice were vaccinated on weeks 0, 3, and 6. Four-weeks after the last boost, we conducted nociception tests using tail flick and hotplate assays in mice using challenges with 30 and 100 μg/kg fentanyl during weeks 10–12 (Fig. 6a). There was a one-week washout period between FEN test doses. These tests were chosen to evaluate the efficacy of vaccines in reducing opioid-induced brain and spinal antinociception effects to provide a measure of vaccine potency. Both assays have been extensively used in identification of lead opioid vaccines^12,13^. All mice developed serum anti-FEN IgG antibodies at weeks 6, 8, and 10; the highest levels of anti-FEN IgG were observed in the alum IM, 1 μg dmLT, and LTA1 IN groups (Fig. 6b). Protection from FEN-induced antinociception was greatest in the LTA1 IN group, followed by the dmLT SL and 1 μg dmLT IM groups (Fig. 6c, d). We observed similar protection from FEN brain penetration, with the majority of injected FEN remaining in the serum unable to penetrate the CNS (Fig. 6e). While FEN brain distribution and serum IgG antibodies at week 8 were significantly correlated (Fig. 6f), protection from antinociception (tail flick assay during 100 μg/kg fentanyl challenge) was not. This may be attributed to a limited sample size or the spinal cord mechanism of tail flick analgesia and our measurement of brain, but not spinal cord levels of FEN, which can differ. Regardless, these studies reveal that both dmLT and LTA1 adjuvant by parenteral and mucosal delivery provide protection against FEN challenge.

### Protection from FEN challenge is strongly correlated to anti-FEN serum IgA, as well as anti-FEN serum IgG binding affinity and IgG2a

**Fig. 7:**
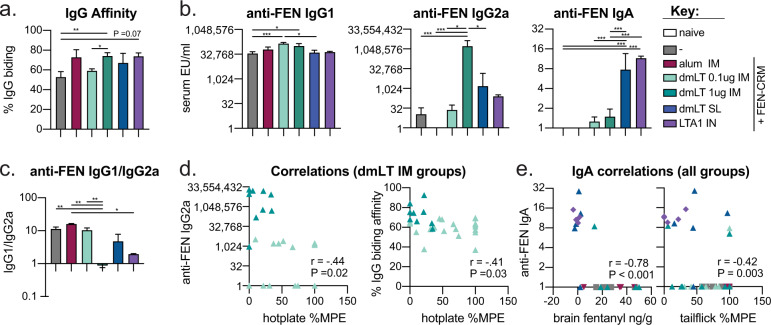
Anti-FEN antibody affinity, IgG2a, and IgA isotypes contributed to protection from fentanyl challenge in dmLT and LTA1 immunized animals. Serum samples from fentanyl challenge experiments were further characterized for anti-FEN immunity. **a** IgG antibody binding affinity calculated as % IgG binding to FEN-TT in the presence of a chaotropic agent (week 6). **b** Compiled serum anti-FEN (FEN-TT) serum IgG1 (week 8), IgG2a (week 8), IgA (week 10) ELISA from tailbleeds. **c** Ratio of serum anti-FEN (FEN-TT) IgG1/IgG2a. (Zero values for IgG2a were replaced with a 1.) **d** Correlation between hotplate antinociception vs IgG2a or IgG binding affinity for 0.1 and 1 μg dmLT IM immunization groups only. **e** Correlation between brain fentanyl levels or tail flick antinociception %MPE vs anti-FEN serum IgA (week 10) for immunized groups. Bars at mean + s.e.m. with significance determined by ANOVA paired with Bonferroni post-hoc test as shown (*P < 0.05, **P < 0.01, ***P < 0.001). Spearman correlations *P* values and correlation coefficient (*r*) indicated on relevant graph.

To determine if we could better understand the correlates of protection in our challenge model, we evaluated serum from our protection study (e.g., Fig. 7) for anti-FEN IgG antibody affinity and antibody isotypes (IgG1, IgG2a, IgA). Both antibody isotypes and binding affinity can improve drug binding and sequestration of opioids from the brain^63^. We observed that both adjuvant selection and route impacted the quality of the antibody responses to FEN (Fig. 7a–c). In particular, the alum group resulted in only anti-FEN IgG1, whereas dmLT and LTA1 adjuvant groups also resulted in IgG2a with maximal levels in the 1 μg dmLT IM group. The group that received alum also had significantly skewed serum IgG1/IgG2a ratio (Fig. 7c). As seen previously highest levels of anti-FEN IgA were observed in mucosal booster groups, including dmLT IM+SL+SL and LTA1 IM+IN+IN. Next, we correlated these anti-FEN antibody affinity and isotype responses from immunized groups to each FEN challenge outcome using Spearman’s correlation test (Supplemental Fig. 4). Anti-FEN IgG2a shows moderate correlation to serum FEN post-challenge (*P* = 0.07, *r* = 0.38), and IgG binding affinity demonstrated a modest inverse relation with % MPE hotplate antinociception (*P* = 0.06, *r* = −0.28). Improved anti-FEN IgG2a isotype class switching and IgG binding affinity appeared to be the major driver of protective responses in higher vs lower dose dmLT IM immunizations with the expected inverse relationship to %MPE (respectively, *P* = 0.02 and *r* = −0.44 or, *P* = 0.03 and *r* = −0.41 Fig. 7d). In contrast, anti-FEN IgA was significantly related to all FEN challenge outcomes measured (*P* = 0.03 to <0.001), including brain fentanyl (*r* = −0.78) and tail flick antinociception (*r* = −0.42; Fig. 7e and Supplemental Fig. 4). These results confirm the importance of IgG2a isotype, but also reveal a previously unknown role for anti-FEN IgA in FEN analgesic effects, which was maximally induced by mucosal boosting with LTA1 IN or dmLT SL vaccination.

## Discussion

An effective FEN vaccine could provide a valuable immunotherapy to attenuate FEN’s physiological, behavioral, and reinforcing effects by preventing the drug from penetrating the CNS. To achieve this, vaccine studies have strived to produce large quantities of FEN-specific antibodies with high binding affinity. In this study, we found that FEN-carrier vaccination with combinations of dmLT and LTA1 adjuvants delivered by IM, IN, or SL routes produced high antibody titers and had significant protective effects against FEN challenge, superior to alum adjuvant. This is the only report of any enterotoxin-derived adjuvant improving efficacy of an opioid vaccine or any substance abuse vaccine. This is also the only report identifying mucosal booster vaccination and generation of IgA antibody isotypes having a significant role in the efficacy of an opioid vaccination strategy.

Administration of the FEN-CRM vaccine with dmLT or LTA1 using IM delivery evoked a potent and long-lasting immune response, with sufficient antibody levels lasting six weeks post-immunization to produce significant blockade of FEN behavioral effects. In spite of being smaller than the typical BSA carrier and having only a lower haptenization ratio, CRM_197_ was an effective carrier protein, showing results similar to recent studies using CRM_197_ for a morphine or oxycodone vaccine^20,26^ and recently for FEN vaccination^64^. Minor differences were observed between dmLT and LTA1 depending upon number of booster immunizations and specific analyses performed (Figs. 2–3); however, these adjuvants were consistently superior to the alum adjuvant comparison groups (Figs. 2, 3, 6, and 7). Both of these adjuvants broadened the immune response to target the FEN hapten more rapidly (e.g., after one booster) than the more commonly used alum adjuvant^12,13,23,25^, and alum generated more rapid and higher responses to the carrier protein antigen BSA or CRM_197_. We chose dmLT IM for challenge experiments, given dmLT’s advanced status in clinical trials^36^ and slightly higher levels of anti-FEN IgG ASCs after three doses (Fig. 3). We observed greater protection from FEN challenge with 1 μg dmLT over 0.1 μg dmLT. This robust attenuation of FEN’s analgesic effects was likely driven by the improved FEN antibody binding and induction of IgG2a isotypes in the higher dose group (Fig. 7). IgG2a has been previously identified as superior to IgG1 in protection against opioids, including a morphine vaccine in mice depleted or lacking in IL-4 cytokine, which reduced class switching to IgG1 with alum adjuvanted vaccination^63^. In conclusion, dmLT may be a superior adjuvant for opioid use disorder when multiple immunizations are provided by parenteral injection due to higher IgG2a isotype generation and FEN antibody binding.

Given the nature of dmLT and LTA1 adjuvants, we also tested mucosal booster immunizations. dmLT has successfully been delivered by sublingual vaccination^43,52,58,61,62^ and LTA1 by intranasal vaccination^39,48^. In addition, within the intended recipient population of a FEN vaccine, many individuals take daily buccal buprenorphine (a pill dissolving between the gums and the cheek) to manage opioid dependence relapse and cravings^65^. The addition of a self-delivered mucosal or intranasal booster vaccine within this treatment context could be advantageous. We observed high levels of anti-FEN immunity in the animals receiving mucosal boosters, including dmLT SL or LTA1 IN, as well as the best blockade of FEN analgesia and brain tissue distribution. The LTA1 IN group achieved the best overall protection; however, despite these responses, anti-FEN IgG, IgG1, IgG2a, and antibody binding affinity were similar to the adjuvanted IM booster groups (Figs. 6–7). One difference we were able to identify was induction of anti-FEN IgA in these mucosal booster groups. IgA levels also correlated to protection from parenteral FEN challenge better than any other antibody measure assayed and this relationship was dose-dependent (Figs. 6f and 7e, Supplemental Fig. 4). At this point, it is unclear if serum IgA or secretory IgA (unmeasured) play direct roles in protection from challenge or if serum IgA is just a surrogate of another protective responses. Regardless, this is the only such report of IgA related to opioid vaccination and drug protection. IgA antibodies have been reported after cocaine and nicotine intranasal vaccines that protected animals from corresponding drug challenges^28,66,67^. IgA has also long been appreciated for high affinity binding to toxins and microbes at mucosal tissue, where most IgA secreting plasma cells reside. This is likely the case in our study, where serum IgA level did not correlate to IgA ASC in the bone-marrow (Fig. 5c). It is interesting to note that IgA is also found in cerebral spinal fluid of healthy humans in both monomeric and dimeric forms^68^. Future evaluations into if or how IgA could be more protective against FEN parenteral challenge (or smoked FEN inhalation) than IgG subclasses are warranted to better understand this phenomenon.

In our ELISA analyses, we observed strong detection of anti-FEN antibodies through FEN-BSA or FEN-TT conjugate antigens, but with slight variability between assays (Fig. 5). Serum IgG levels against FEN were higher with FEN-TT than with FEN-BSA coating antigens, indicating that quantification of anti-FEN immunity must be considered in light of the differences in sensitivity and quantification observed in assays using carrier antigens as detection systems for antibodies. For example, SL dmLT groups had more appreciable levels of anti-FEN serum IgA when FEN-TT was used as coating antigen than with FEN-BSA coating antigen (Figs. 4 and 7, Supplemental Fig. 3). In addition, we reported that serum IgG (by ELISA) significantly correlated with the number of IgG-secreting ASCs found in the bone-marrow, both of which were maximally enhanced in dmLT or LTA1 adjuvant groups. Similarly, flow cytometry studies have shown that ASC B cells at 14 days post immunization correlated with vaccine efficacy against opioids, with adjuvant-dependent induction of these ASC and germinal center formation (where B-cell clonal expansion, differentiation, and maturation take place)^63,69^.

In conclusion, we report that dmLT and LTA1 adjuvants enhance immunogenicity of an anti-FEN conjugate vaccine and promote a robust blockade of FEN-induced analgesia. We observed strong evidence for a greater protective effect with vaccination that included mucosal booster immunizations with subsequent induction of IgA. Future investigation and development of a FEN vaccine with these adjuvants and combinatorial or mucosal delivery routes are warranted, including the duration of vaccine-mediated blocking of FEN analgesia, respiratory depression, other toxicity or reinforcing effects and comparisons or combinations with current FDA-approved pharmacotherapies (e.g., buprenorphine). Furthermore, in light of our findings we anticipate that combination of a dmLT or LTA1 opioid vaccination with antigens for other substances associated with use disorders, including cocaine, amphetamines, and nicotine, could benefit the design of new immunotherapies against SUDs.

## Methods

### Antigens and adjuvants

FEN-BSA was purchased from Cal BioReagents. FEN-CRM and FEN-TT were synthesized using a FEN derivative with a carboxylic acid linker coupled to lysine residues on CRM_197_ (Fina Biosolutions) or TT (Statens Serum Institute) similar to previously published^13^. Briefly, the FEN hapten was created in a series of four chemical reactions starting with pure FEN (depicted in Supplemental Fig. 5). The product of each step was characterized and validated by ^1^H and ^13^C NMR spectrum (Supplemental Fig. 6). Purity of the FEN hapten was validated by HPLC (Supplemental Fig. 7). The final product was then conjugated to CRM_197_ as described in Supplemental notes. FEN-CRM characterization by MALDI-TOF gave a haptenization ratio of 2.3. All conjugates were dialyzed in PBS and quantified using BCA kit (Pierce) prior to immunization. dmLT GLP was produced according to cyclic GMP (cGMP) specification by IDT in sodium phosphate buffer supplemented with 5% lactose as a lyophilized product in vials containing 400 μg product in a 3 ml sterile, multidose, Wheaton serum vial and was stored at 4 °C. His-tagged LTA1 was prepared from solubilized inclusion bodies by HPLC with a nickel-affinity column as previously described^39^. Proteins were stored lyophilized and freshly resuspended prior to use (dmLT) or kept frozen at −80 °C until use (LTA1). Alum (Alhydrogel^®^ adjuvant 2%) was purchased from Invitrogen.

### Animals, immunizations, and sample collections

Female BALB/c mice, 6–8 weeks of age, were purchased from Jackson Laboratories or Charles River (challenge experiments) and housed in sterilized cages. Animal studies were approved by Tulane University and University of Houston Institutional Animal Care and Use Committee. Immunization formulations were prepared immediately before administration by admixing antigen +/− adjuvant in sterile PBS in a 20 μl volume or absorbing to alum in 20–100 μl volume. Animals were injected with a 0.5cc insulin syringe into the right or left caudal thigh muscle, alternating sides with each intramuscular immunization (or both legs for 1500 μg dose alum/50 μl per leg, note that this dose of alhydrogel or Al(OH)3 has a ~3.3x greater weight than free Al3+ and thus is well below the recommended human doses of 1.14 mg Al3+ (US Code of Federal Regulations, 21CFR610.15)). For intranasal immunizations, mice were first anesthetized with intraperitoneal (IP) ketamine/xylazine then kept horizonal while formulations were pipetted into one or both nostrils. For sublingual immunizations, mice were anesthetized with IP ketamine/xylazine and then held with their jaw horizontal lifting up their tongue with forceps while formulations were pipetted under the tongue. This position was held for 1–2 min, then the mouths of the mice were closed and held for another min. Immunizations were performed 2 or 3 times at 3-week intervals prior to CO_2_ euthanasia for sample collection or FEN challenge. Blood was collected by tail or cardiac venipunctures and processed for serum. Spleens were homogenized in 3 ml of PBS containing 2% BSA (MilliporeSigma) and 1 mM EDTA using gentleMACS C tubes and tissue dissociator (Miltenyi Biotec). Splenocytes were then RBC lysed using ACK lysis buffer (Gibco), filtered, and counted. Both left and right tibia and fibulas were removed, stripped of muscle and connective tissue, and bone marrow was flushed using 10 ml of PBS containing 2% BSA and 1 mM EDTA with a 30-gauge needle attached to a 10 ml syringe. Cells were filtered and counted.

### Antibody ELISAs

Anti-FEN, -CRM, and -BSA serum antibody IgG ELISAs were performed using similar methods described in ref. ^57^. Corning 96 well flat bottom plates (Costar 9018) were coated with 0.1 µg CRM197, 0.1 µg BSA, 0.2 µg FEN-BSA, 0.2 µg FEN-TT and detected using AKP-conjugated anti-mouse IgG, IgM (Sigma), IgG1 or IgG2a (BD Biosciences) or HRP-conjugated anti-mouse IgA (Sigma). ELISAs were quantified with dilutions of purified mouse standards IgG1-κ, IgG2a-κ, IgM-κ, or IgA-κ (Sigma). Results were expressed as ELISA units/ml (EU/ml) using an average of two sample dilutions closest to the midpoint of the standard curve and graphed on a log2 scale. For binding affinity ELISAs, the IgG serum ELISA procedure from above was modified by testing two dilutions of sera per sample in duplicate (from linear range based on IgG ELISA or 1:1600 – 1:12800 dilutions). Plates were developed for IgG after 1 h serum incubation, 6x plate washing, 15–20 min 0, 2, 4, 6M ammonium thiocyanate, and 3x plate washing. Affinity was quantified as % IgG binding using ratio of OD values from 0M/2M wells.

### ELISPOT ASC and memory B cells

ELISPOTS were performed following the ImmunoSpot B-Cell Double-Color Enzymatic Mu B-Cell IgA/IgG ELISPOT kit (ImmunoSpot). ASC plates were coated with 0.1 ug/well of FEN-TT. After 24h, splenocytes and bone marrow cells were plated in duplicate dilutions at 4 different concentrations starting from 0.5, 1.0, or 2.5 x 10^6^ cells and incubated at 37 °C for 24 h. Kit instructions were followed for detection, with overnight incubation of the Anti-Murine IgG/IgA detection solution and 20 min incubation with Blue and Red Developer Solutions. Plates were dried overnight and imaged with ImmunoSpot S5 Macro Analyzer with ImmunoCapture 6.3.3. Spots were quantified with ImmunoSpot software. For memory B-cell analysis, cells were adjusted to 4 x 10^6^ cells/ml in a 24-well non-tissue culture treated plate (Falcon) and incubated for 4 days at 37 °C in CTL-Test B culture medium with 1% L-Glutamine. Pre-stimulated cells were harvested after 4 days, recounted, diluted, and plated in the same manner as ASCs above.

### FEN challenge and nociception tests

Fentanyl citrate salt was purchased for these studies (Sigma-Aldrich). Nociception tests were conducted using tail flick and hotplate assays^70–72^ during weeks 9–12. Tail flick tests were conducted by restraining the mouse using a device that enabled a consistent angle so the tail could be exposed to an infrared heat stimulus (25 IR) 3 cm from the tip using an automated device (Ugo Basile). The time from the onset of the heat to the withdrawal of the tail (latency) was automatically recorded by the machine. Baseline latencies were first recorded following saline administration and were determined three times (1–2 min inter-trial interval). Mice were then administered one of two doses of FEN (30 and 100 μg/kg, IP) in a counterbalanced order with at least one week intervening between tests. Latencies were determined at 10 min post injection. Three measures were acquired with an inter-trial interval of 1–2 min. A single hot plate test was conducted immediately after the tail flick tests using a hotplate apparatus (Columbus Instruments, Columbus, OH). To control for baseline differences following saline, data from the tail flick assay are presented as %MPE (percent Maximum Possible Effect) − (test latency−control latency)/(cutoff criterion−control latency) × 100. Hot plate data are presented as mean (+s.e.m.) latency. A cut off time of 10 s for tail flick and 90 s for hotplate was used to prevent tissue damage (cut-off latency). Brain and blood samples were collected as done previously^73^. For the final blood and tissue collection, mice were administered 100 μg/kg fentanyl and anesthetized with isoflurane. Ten minutes later, mice were deeply anesthetized with isoflurane anesthesia. A bilateral thoracotomy was performed and whole blood removed via left ventricle puncture with a 22-gauge needle then processed for serum. The heart was perfused with cold PBS then the brain was removed, washed in PBS, and immediately placed on dry ice. Samples were stored at −80 °C until FEN levels were determined. After hot-plate tests, serum and brain were collected and analyzed for FEN tissue analyses.

### FEN tissue level analysis

Samples were diluted in an acetonitrile solution (1:3) and centrifuged at 8609 x *g* for 10 min. The supernatant was transferred, evaporated, and diluted 1:1 in PBS. Samples were extracted using Bond Elut Plexa PCX, 3mL extraction cartridges (Agilent), evaporated, and reconstituted in a solution of H_2_O:0.1% ammonium formate:0.01% formic acid. Sample were injected onto a reversed phase Agilent Zorbax Eclipse plus C18 column (2.1 mm × 50 mm i.d., 1.8 μm). The LCMS/MS system consisted of an Agilent G6470A TQ with an Infinity II 1290 G7116B Multicolumn Thermostat, G7120A High Speed Quad Pumps, G7267B Multisampler. Data were analyzed using Mass Hunter software.

### Western blot

Protein conjugates and controls were analyzed by Western blot. Cell lysates were loaded into NuPage 12% or 4–12% Bis-Tris gel wells (ThermoFisher Sci.) for gel electrophoresis, then transferred to nitrocellulose membrane using iBlot Transfer Stacks and the iBlot Gel Transfer Device (ThermoFisher Sci.). Blots were initially stained with Ponceau stain, then blocked 5% skim milk and probed with anti-Fentanyl (Cal BioReagents) and goat anti-mouse IgG1-HRP (Santa Cruz). After imaging, blots were stripped with Restore Plus Stripping Buffer (ThermoFisher Sci.) then re-developed with mouse anti-CRM antibody (Antibody and Immunoassay Consultants) and goat anti-mouse HRP antibody (Santa Cruz). Blots were imaged with Pierce™ ECL Western Blotting Substrate (ThermoFisher Sci.) and Amersham Imager 600.

### Statistical analysis

Statistical analyses were performed using Prism (GraphPad Software v7). Parametric data were analyzed by one-way ANOVA with Dunnett’s post-test for all compared to a control group or Bonferroni correction for comparison of selected pairs. F-statistics, degrees of freedom, and significance are also recorded in Supplemental Table 1. Data were tested to confirm lack of normality (D’Agostino & Pearson) and then tested by Spearman correlation.

### Reporting summary

Further information on research design is available in the Nature Research Reporting Summary linked to this article.

## Supplementary information

Supplementary Information

Reporting Summary

## Data Availability

The datasets generated and/or analyzed in this study (and its Supplementary Information files) are available from the corresponding author on reasonable request.
