# Supplementary material for: Fentanyl conjugate vaccine by injected or mucosal delivery with dmLT or LTA1 adjuvants implicates IgA in protection from drug challenge
Source: NPJ Vaccines. 2021 May 13;6:69. doi: 10.1038/s41541-021-00329-0 (PMC8119695; doi:10.1038/s41541-021-00329-0)
Supplement: Supplementary file 1 — Supplementary Information [file 41541_2021_329_MOESM1_ESM.pdf]

SUPPLEMENTAL INFORMATION: Novel Fentanyl Conjugate Vaccine by Injected or Mucosal Delivery with dmLT or LTA1 Adjuvants Implicates IgA in Protection from Drug Challenge

SUPPLEMENTAL FIGURES

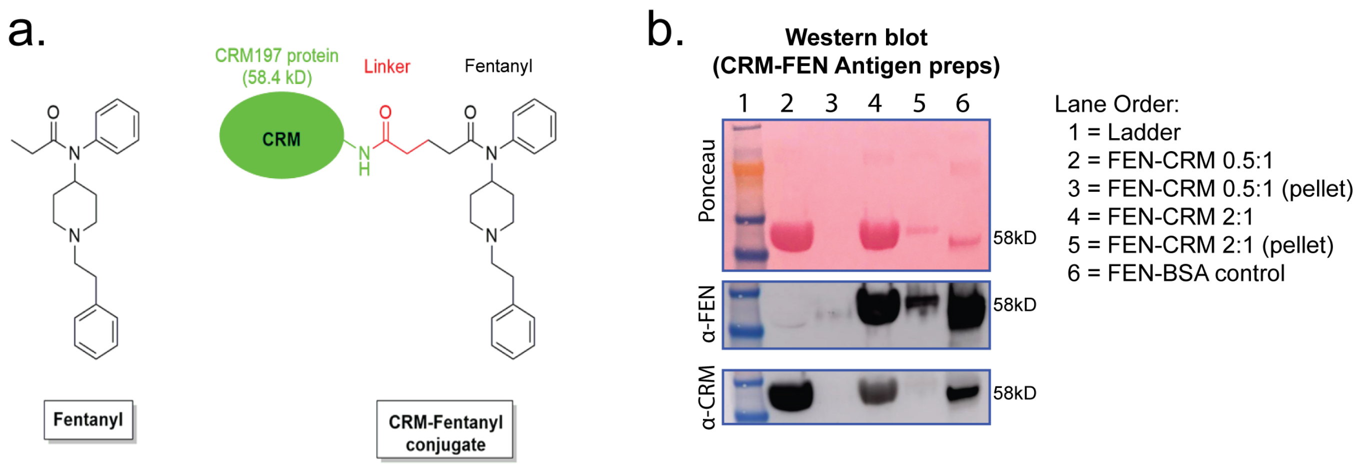

Supplemental Figure 1. Preparation of FEN-CRM conjugate protein. (A) Depiction of fentanyl derivative containing a linker with a carboxyl moiety suitable for carbodiimide coupling chemistry to form amide bonds to lysine residues on CRM_{197}. (B) Representative Western blot staining of FEN-CRM conjugates synthesized at 0.5:1 or 2:1 hapten to CRM_{197} ratios compared to FEN-BSA control. Sample was microcentrifuged prior to gel loading to obtain supernatant or pellet. Blot was evaluated by Ponceau stain for total protein, anti-FEN staining, then stripped and evaluated for anti-CRM staining as shown (same blot from a single representative experiment is shown that was processed sequentially). Two blue ladder bands correspond to 62kD and 49kD. The 2:1 hapten ratio was the most optimal to keep protein in solution rather than precipitating as a pellet, with higher levels of anti-FEN staining compared with the 0.5:1 hapten ratio and was selected for all subsequent vaccination experiments.

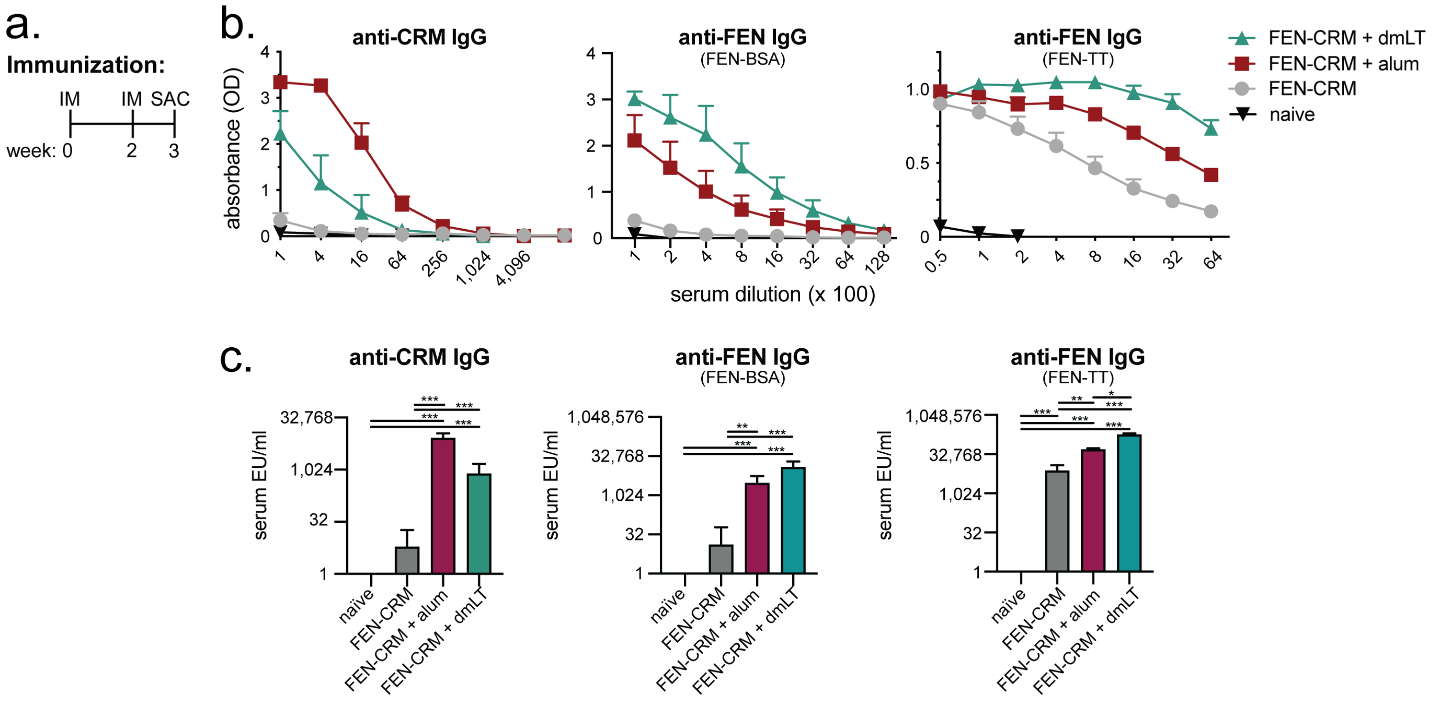

Supplemental Figure 2. Immune responses to prime/boost intramuscular FEN-CRM197 immunization are enhanced in magnitude with dmLT adjuvant. Groups Balb/c mice (n=5) were left naïve or immunized on weeks 0 and 2 with 1.5μg BSA-FEN alone or combined admixed with 1μg dmLT or with absorption 1:1 with 2% alyhydrogel (alum, 100μg final dose) and delivered intramuscularly in 20 μl. Serum was colleted for ELISAs on week 3. (A) Schematic of immunization. (B) Raw ELISA data from serum anti-CRM or FEN serum IgG (coating antigen indicated) expressed as absorbance. (C) Compiled EU/ml of indicated serum anti-CRM or FEN serum IgG (coating antigen indicated). Bars at mean + s.e.m. with significance determined by ANOVA paired with Bonferroni post-hoc test as shown (*$P < 0.05$, ** $P < 0.01$, *** $P < 0.001$).

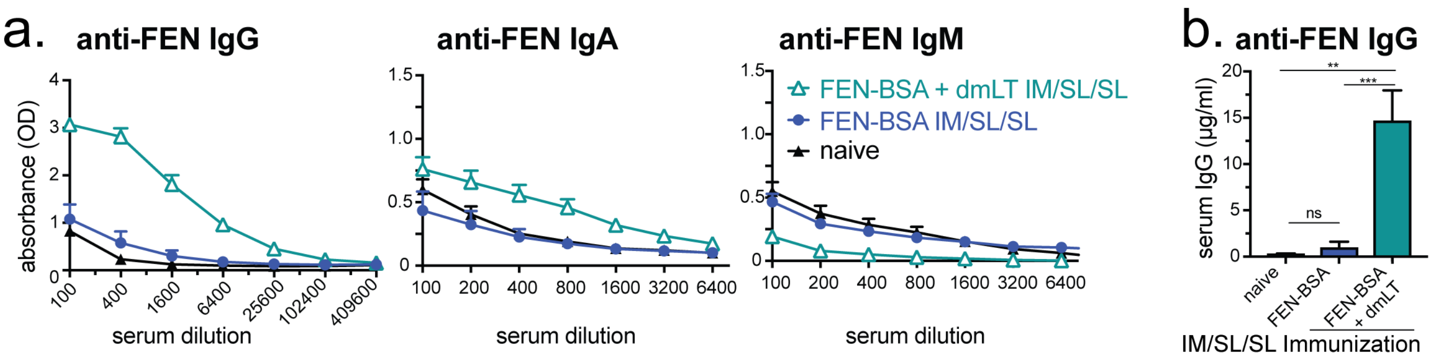

Supplemental Figure 3. Sublingual booster vaccinations help induce serum IgG and serum IgA to fentanyl. Groups Balb/c mice (n=8) were immunized on day 0 with 8µg FEN-BSA alone or combined admixed with 1µg dmLT intramuscularly in 20 µl, then boosted on days 21 and 42 with 10µg BSA-FEN alone or combined admixed with 5µg dmLT administered sublingually in 12µl by pipetting underneath the tongue of anesthetized mice. Anti-fentanyl serum IgG, IgA, and IgM antibody levels were analyzed on day 56 by ELISA plated with FEN-TT in comparison with naïve mouse sera with raw data (A) and anayzed data (B) shown. Bars at mean + s.e.m. with significance indicated as ns = not significant, ** $P<0.01$, *** $P<0.001$ by ANOVA paired with Bonferroni post-hoc test.

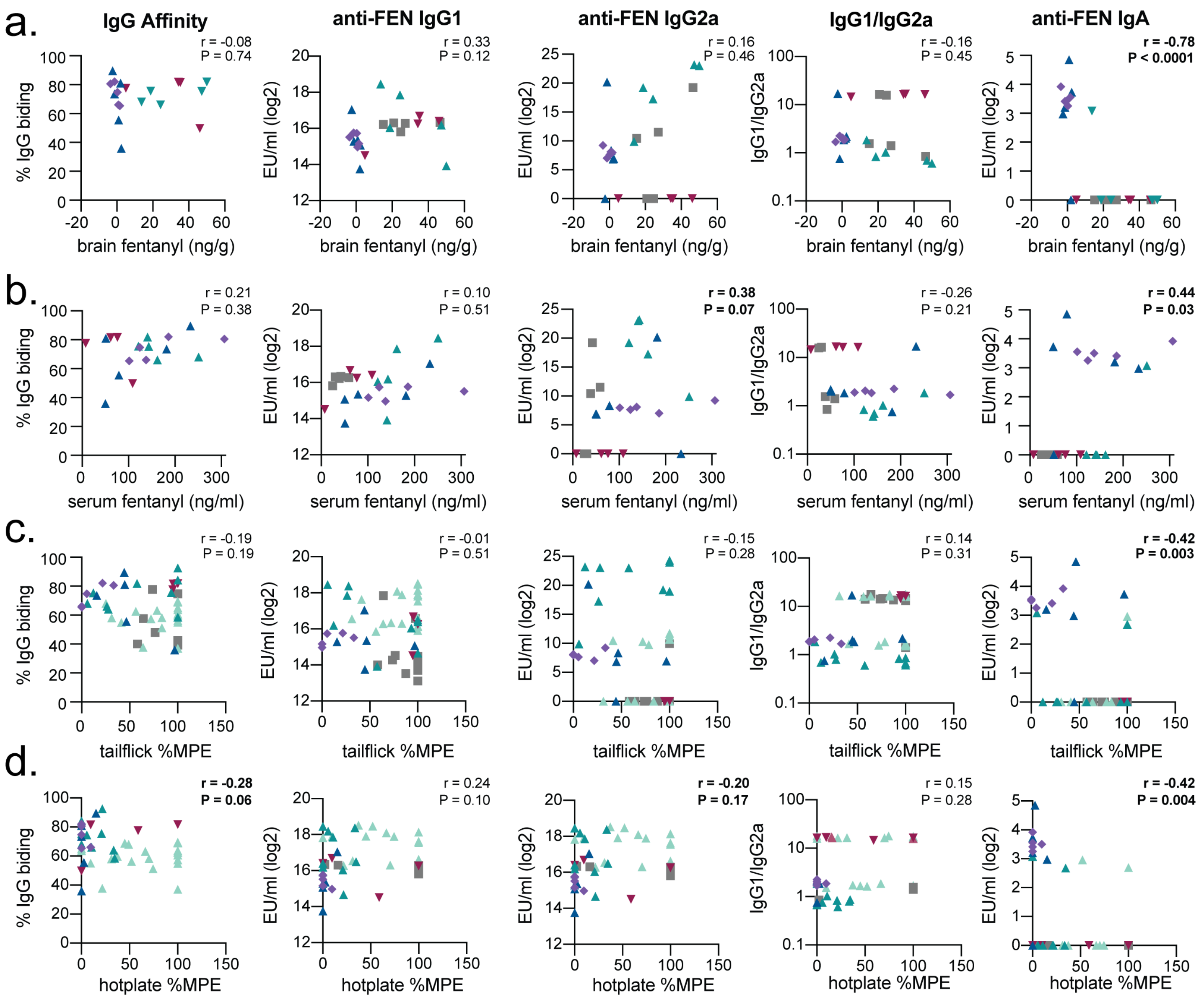

Supplemental Figure 4. Correlations between anti-FEN antibody analyses and FEN challenge assays. Anti-FEN serum ELISAs (week 6-10) results were correlated to week 9-12 fentanyl challenge outcomes. Correlation for immunized animals between IgG antibody binding affinity (week 6), IgG1 (FEN-TT, week 8), IgG2a (FEN-TT, week 8), IgG1/IgG2a ratio (week 8), or IgA (FEN-TT, week 10) and (A) brain fentanyl levels, (B) serum fentanyl levels, (C) 100 µg/kg tailflick antinociception %MPE, or (D) 30 µg/kg hotplate antinociception %MPE. Spearman correlations P values and correlation coefficient (r) indicated on relevant graph. Symbol colors indicate immunization group from Figure 6 & 7. EU/ml are shown as log2 transformed (e.g, a value of 1 on the graph = 2; 5 = 32; 10 = 1,024; 15 = 32,768; 20 = 1,048,576).

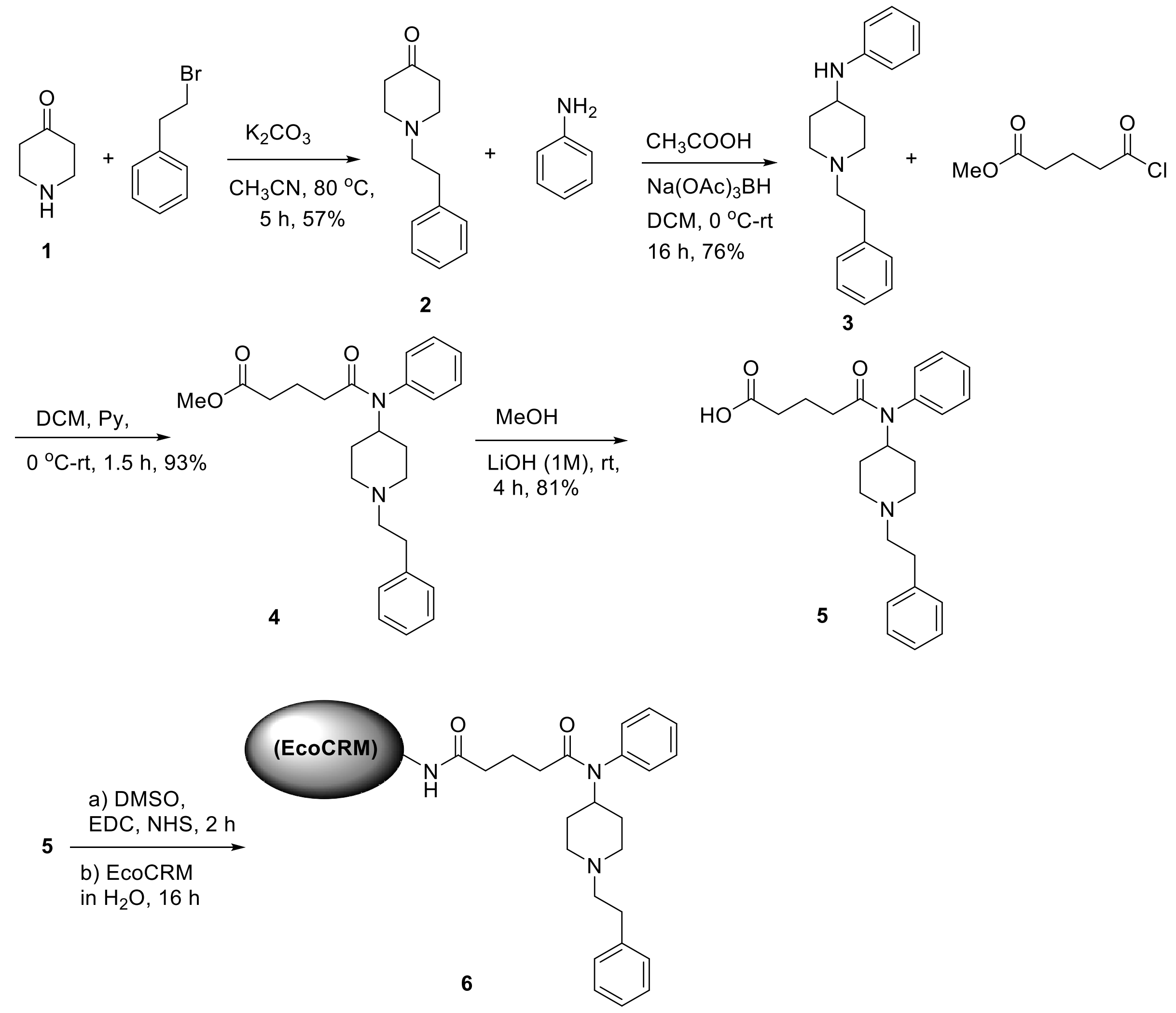

Supplemental Figure 5. Steps in synthesis of Fentanyl-CRM conjugate.

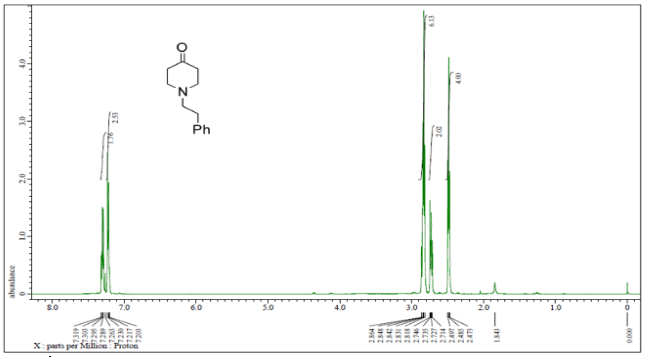

^{1}H NMR spectrum of **2** ($CDCl_3$, 500 MHz)

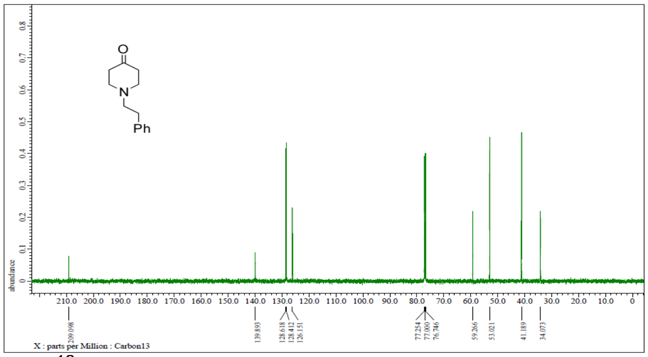

^{13}C NMR spectrum of **2** ($CDCl_3$, 125 MHz)

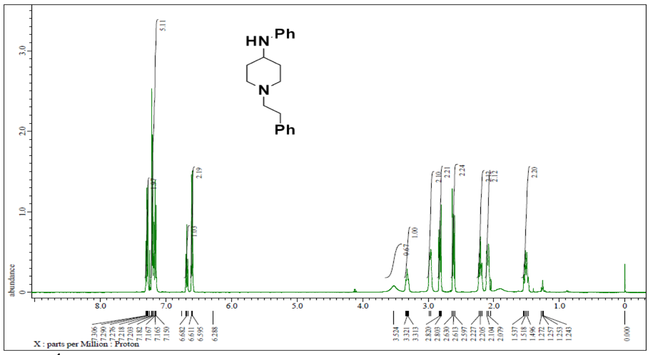

^{1}H NMR spectrum of **3** ($CDCl_3$, 500 MHz)

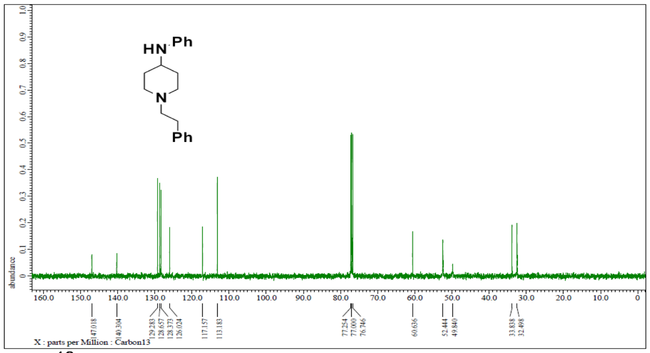

^{13}C NMR spectrum of **3** ($CDCl_3$, 125 MHz)

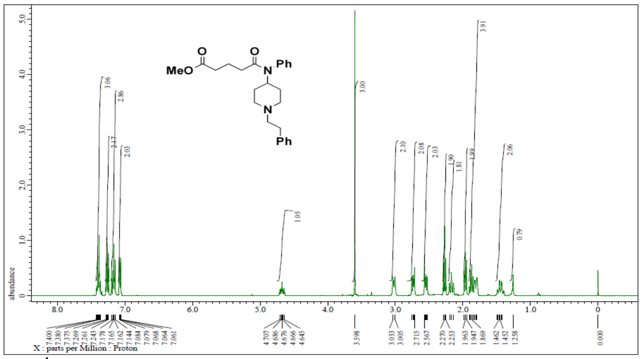

^{1}H NMR spectrum of **4** ($CDCl_3$, 400 MHz)

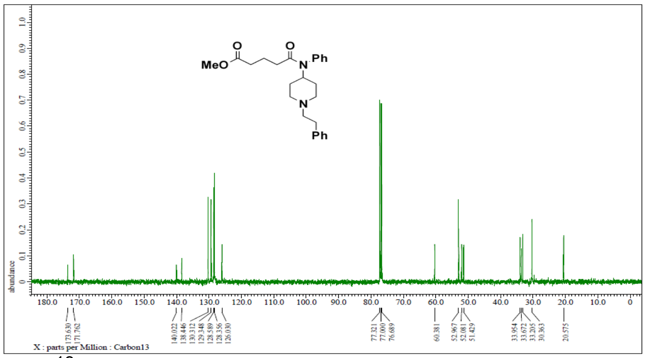

^{13}C NMR spectrum of **4** ($CDCl_3$, 100 MHz)

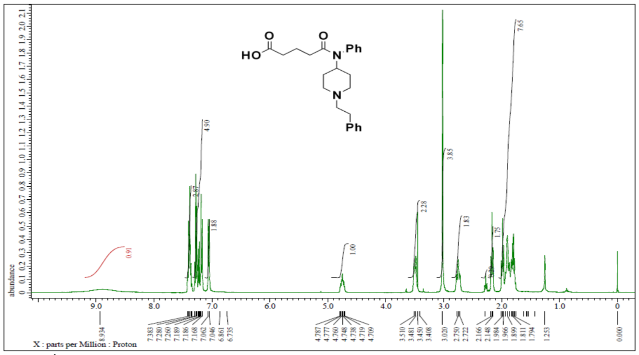

^{1}H NMR spectrum of **5** ($CDCl_3$, 400 MHz)

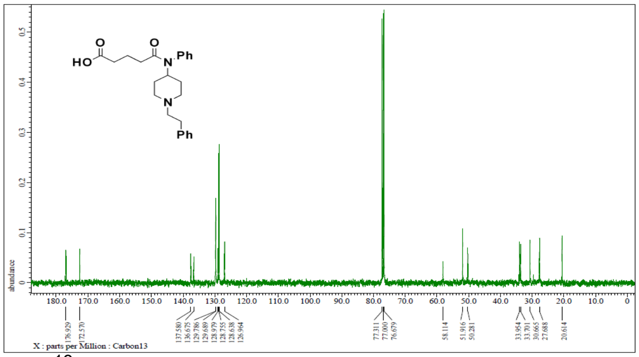

^{13}C NMR spectrum of **5** ($CDCl_3$, 100 MHz)

Supplemental Figure 6. **^{1}H and ^{13}C NMR spectra.** The product of each step during FEN hapten creation was characterized and validated by ^{1}H and ^{13}C NMR spectrum.

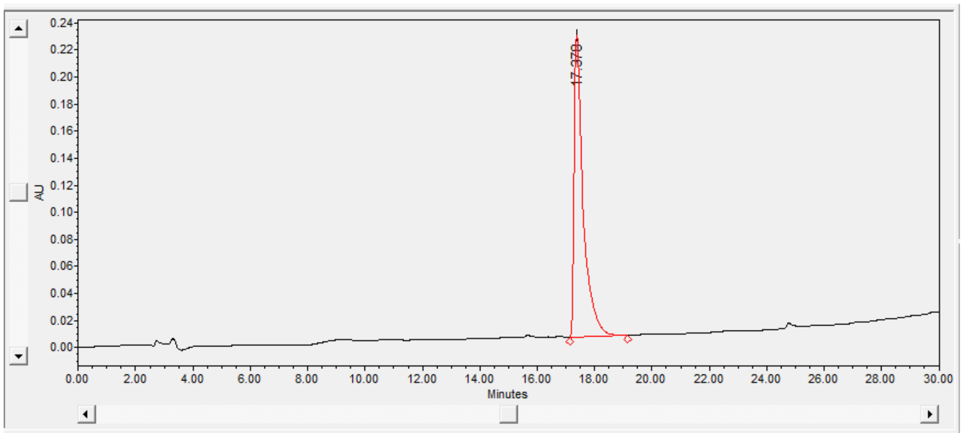

	Name	Retention Time (min)	Area (µV*sec)	% Area	Height (µV)	Int Type	Amount	Units	Peak Type	Peak Codes
1		17.378	4938919	100.00	223108	VV			Unknown	

3D Channels | 2D Channels | Peaks

Supplemental Figure 7. HLPC Analysis of hapten 5

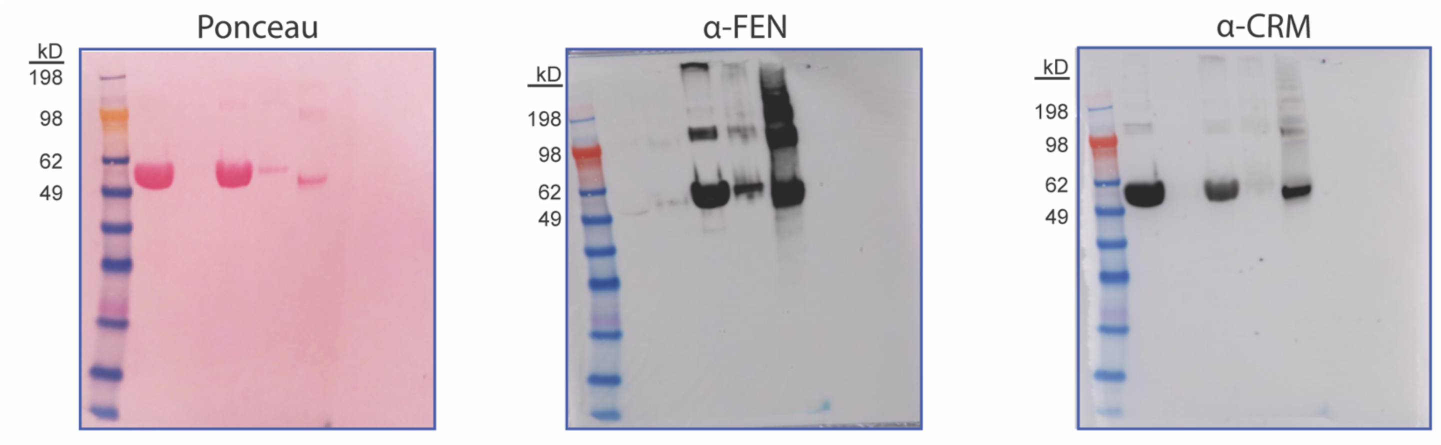

Supplemental Figure 8. Complete blot images (from Supplemental Figure 1) with SeeBlue Plus 2 ladder Molecular Weights indicated.

Supplemental Table 1. F-statistics and degrees of freedom for each manuscript figure.

Figure	Assay Evaluated	F (DFn, DFd)* = F statistic	P value
Fig 1C	anti-BSA IgG	F (2, 7) = 254.6	P<0.0001
Fig 1C	anti-FEN-TT IgG	F (2, 7) = 80.01	P<0.0001
Fig 1D	anti-FEN-TT IgG1	F (2, 7) = 302.3	P<0.0001
Fig 1D	anti-FEN IgG2a	F (2, 7) = 337.7	P<0.0001
Fig 1G	anti-FEN ASC	F (3,12) = 71.46	P<0.001
Fig 2C	anti-CRM IgG Week 5	F (4, 18) = 9.820	P=0.0002
Fig 2C	anti-CRM IgG Week 9	F (4, 18) = 101.1	P<0.0001
Fig 2C	anti-FEN-BSA IgG Week 5	F (4, 18) = 10.83	P<0.001
Fig 2C	anti-FEN-BSA IgG Week 9	F (4, 18) = 4.453	P=0.011
Fig 2C	anti-FEN-TT IgG Week 5	F (4, 18) = 91.96	P<0.0001
Fig 2C	anti-FEN-TT IgG Week 9	F (4, 18) = 145.0	P<0.0001
Fig 2E	anti-FEN ASC Week 5	F (4, 16) = 20.14	P<0.0001
Fig 2E	anti-FEN ASC Week 9	F (4, 18) = 7.455	P=0.0010
Fig 2F	anti-FEN memory B cells BM	F (4, 16) = 6.130	P=0.0035
Fig 2F	anti-FEN memory B cells spleen	F (4, 18) = 0.8560	**P=0.5087**
Fig 3B	anti-CRM IgG	F (4, 17) = 205.2	P<0.0001
Fig 3B	anti-FEN-TT IgG	F (4, 17) = 142.0	P<0.0001
Fig 3B	anti-FEN-BSA IgG	F (4, 17) = 10.67	P=0.0002
Fig 3C	anti-FEN-BSA IgG1	F (4, 17) = 9.088	P=0.0004
Fig 3C	anti-FEN-BSA IgG2a	F (4, 17) = 3.146	P=0.0416
Fig 3E	anti-FEN ASC IgG	F (4, 17) = 9.653	P=0.0003
Fig 4B	anti-CRM IgG	F (4, 22) = 63.28	P<0.001
Fig 4B	anti-FEN-BSA IgG	F (4, 22) = 14.10	P<0.001
Fig 4B	anti-FEN-TT IgG	F (4, 22) = 33.94	P<0.0001
Fig 4B	anti-FEN-BSA IgA	F (4, 22) = 5.316	P=0.0038
Fig 4C	anti-FEN-TT IgG1	F (4, 22) = 80.05	P<0.0001
Fig 4C	anti-FEN-TT IgG2a	F (4, 22) = 7.677	P=0.0005
Fig 4E	anti-FEN ASC IgG	F (4, 22) = 5.827	P=0.0024
Fig 4E	anti-FEN ASC IgA	F (4, 22) = 0.2831	**P=0.8858**
Fig 6B	anti-FEN-TT IgG Week 6	F (5, 51) = 8.270	P<0.0001
Fig 6B	anti-FEN-TT IgG Week 8	F (5, 53) = 6.565	P<0.0001
Fig 6B	anti-FEN-TT IgG Week 10	F (5, 50) = 6.315	P=0.0001
Fig 6C	%MPE tailflick (FEN 30ug/kg)	F (6, 54) = 1.659	**P=0.1491**
Fig 6C	%MPE tailflick (FEN 100ug/kg)	F (6, 56) = 10.43	P<0.0001
Fig 6D	%MPE hotplate (FEN 30ug/kg)	F (6, 62) = 5.569	P=0.0001
Fig 6D	%MPE hotplate (FEN 100ug/kg)	F (6, 61) = 189.9	P<0.0001
Fig 6E	brain fentanyl ng/g	F (5, 26) = 8.813	P<0.0001
Fig 6E	serum fentanyl ng/ml	F (5, 26) = 9.347	P<0.0001
Fig 7A	IgG Affinity (FEN-TT)	F (5, 46) = 4.402	P=0.0023
Fig 7B	anti-FEN-TT IgG1	F (5, 52) = 6.088	P=0.0002
Fig 7B	anti-FEN-TT IgG2a	F (5, 51) = 17.89	P<0.0001

Fig 7B	anti-FEN-TT IgA	F (5, 54) = 20.95	P<0.0001
Fig 7C	anti-FEN-TT IgG1/IgG2a	F (5, 52) = 7.275	P<0.0001
Suppl Fig 2C	anti-CRM IgG	F (3, 16) = 39.61	P<0.0001
Suppl Fig 2C	anti-FEN-BSA IgG	F (3, 16) = 25.98	P<0.0001
Suppl Fig 2C	anti-FEN-TT IgG	F (3, 15) = 339.8	P<0.0001

*degrees of freedom numerator (dfn); degrees of freedom denominator (dfd)

<u>SUPPLEMENTAL NOTE: Steps for FEN-CRM Conjugatation.</u> The FEN hapten was created in a series of four chemical reactions starting with pure FEN as depicted in Supplemental Figure 5. The product of each step was characterized and validated by ^1H and ^{13}C NMR spectrum (Supplemental Figure 6). Purity of the FEN hapten was validated by HPLC (Supplemental Figure 7). The final product was then conjugated to CRM_{197} as described below. FEN-CRM characterization by MALDI-TOF gave a haptenization ratio of 2.3.

Step 1: ***N-Phenylethylpiperidin-4-one (2)***[1] To a solution of 4-piperidone monohydrate hydrochloride **1** (2.0 g, 14.75 mmol) was dissolved in acetonitrile (40 mL) was added K_2CO_3 (6.1 g, 44.14 mmol) and (2-bromoethyl)benzene (2.46 g, 13.27 mmol) at ambient temperature. The resulting suspension was refluxed at 80 °C for 5 h. The reaction mixture was monitored by TLC (50% ethyl acetate in hexane). Upon completion of the reaction, the reaction mixture was allowed to cool to room temperature and filtered through a small celite pad. The crude material was purified by flash column chromatography (0-30% ethyl acetate in hexane) to afford compound **2** (1.72 g, 57%) as off-white solid; mp 57-59 °C. ^1H NMR (500 MHz, $CDCl_3$): δ 7.32 - 7.29 (m, 2H), 7.23 - 7.20 (m, 3H), 2.86 - 2.82 (m, 6H), 2.75- 2.71(m, 2H), 2.49 (t, *J* = 6.0 Hz, 4H); ^{13}C NMR (125 MHz, $CDCl_3$): δ 209.1, 139.9, 128.6, 128.4, 126.2, 59.3, 53.0, 41.2, 34.1.

Step 2: ***1-Phenethyl-N-phenylpiperidin-4-amine (3)***[2] To a solution of aniline (90 µL, 0.98 mmol) in dichloromethane (3 mL) was added acetic acid (56 µL, 0.98 mmol) drop wise at 0 °C. Subsequently N-phenylethylpiperidin-4-one **2** (200 mg, 0.984 mmol, dissolved in 1 mL dichloromethane) was added drop wise, followed by addition of sodium triacetoxyborohydride (313 mg, 1.476 mmol) in 3 portions over a 12 minutes timeframe. The reaction mixture was stirred at ambient temperature for 16 h. After completion of the reaction, methanol (3 mL) was added and the reaction mixture was partitioned between dichloromethane and sat. $NaHCO_3$.The organic phase was separated and washed with brine, dried over anhydrous Na_2SO_4 and filtered. Solvent was removed under reduced pressure providing the crude compound. This material was purified by flash column chromatography (0-50% ethyl acetate in hexane) to obtain the desired compound **3** as an off-white solid (211 mg,76%). mp 96-98 °C. ^1H NMR (500 MHz, $CDCl_3$): δ 7.31 - 7.28 (m, 2H), 7.22 - 7.15 (m, 5H), 6.68 (t, *J* = 7.5 Hz, 1H), 6.60 (d, *J* = 8.0 Hz, 2H), 3.52 (brs, 1H), 3.34 - 3.30 (m, 1H), 2.98- 2.95 (m, 2H), 2.84 - 2.80 (m, 2H), 2.63 - 2.59 (m, 2H), 2.21 (t, *J* = 10.9 Hz, 2H), 2.10 - 2.07 (m, 2H), 1.54 - 1.46 (m, 2H); ^{13}C NMR (125 MHz, $CDCl_3$): δ 147.0, 140.3, 129.3, 128.7, 128.4, 126.0, 117.2, 113.2, 60.6, 52.4, 49.8, 33.8, 32.5.

Step 3: ***Methyl 5-oxo-5-((1-phenethylpiperidin-4-yl)(phenyl)amino)pentanoate (4)*** To a solution of compound **3** (200 mg, 0.713 mmol) in anhydrous dichloromethane was added pyridine (112 mg, 1.415 mmol) and methyl 4-(chloroformyl)butyrate (129 mg, 0.783 mmol) at 0 °C. The reaction mixture was stirred for 5-10 min at 0 °C. The reaction mixture was allowed to warm to room temperature and then stir for 1.5 h. Reaction progress was monitored by TLC (5% methanol in dichloromethane). Upon completion, the reaction was quenched with saturated $NaHCO_3$ solution. The organic layer was separated and the aqueous layer was washed with ethyl acetate (2 x 10 mL). The combined organic layers were washed with brine, dried over hydrous Na_2SO_4, filtered and concentrated under reduced pressure providing the crude compound, which was purified by flash column chromatography (0-5% methanol in dichloromethane) to obtain the desired compound **4** as a pale yellow viscous liquid (273 mg, 93%). ^{1}H NMR (400 MHz, $CDCl_3$): δ 7.42 - 7.34 (m, 3H), 7.28 - 7.24 (m, 2H), 7.19 - 7.14 (m, 3H), 7.08 - 7.06 (m, 2H), 4.68 (tt, *J* = 12.3 Hz, 3.9 Hz, 1H), 3.60 (s, 3H), 3.03 - 3.0 (m, 2H), 2.75 - 2.71(m, 2H), 2.56 - 2.52 (m, 2H), 2.27 (t, *J* = 7.1 Hz, 2H), 2.20 - 2.14 (m, 2H), 1.98 - 1.94 (m, 2H), 1.91-1.79 (m, 4H), 1.49 - 1.39 (qd, *J* = 12.4 Hz, 3.7 Hz, 2H); ^{13}C NMR (100 MHz, $CDCl_3$): δ 173.6, 171.8, 140.0, 138.4, 130.3, 129.3, 128.6, 128.4, 126.0, 60.4, 53.0, 52.1, 51.4, 34.0, 33.7, 33.2, 30.4, 20.6.

Step 4: ***5-Oxo-5-((1-phenethylpiperidin-4-yl)(phenyl)amino)pentanoic acid (5)*** To a solution of ester **4** (100 mg, 0.244 mmol) in methanol was added 0.5 mL of 1M aqueous LiOH solution. The reaction mixture was stirred for 4 h at room temperature. The progress of the reaction was monitored by TLC (5% methanol in dichloromethane). Upon completion, the reaction solution was acidified with 3N aqueous HCl to pH 5. Then it was concentrated in vacuo to remove methanol. The residue was extracted with ethyl acetate and washed with a small amount of brine solution (note, product is water soluble). The organic layer was dried over anhydrous Na_2SO_4, filtered and concentrated under reduced pressure. The crude compound was purified by flash column chromatography (0-5% methanol in dichloromethane) to yield the target compound **5** as an off-white solid (78 mg, 81%). mp 60-63 °C.^{1}H NMR (400 MHz, $CDCl_3$): δ 8.93 (brs, 1H), 7.42 - 7.34 (m, 3H), 7.30 - 7.16 (m, 5H), 7.06 - 7.04 (m, 2H), 4.78 - 4.70 (m, 1H), 3.51 - 3.48 (m, 2H), 3.02 (s, 4H), 2.77 - 2.72 (m, 2H), 2.17 (t, *J* = 7.1 Hz, 2H), 1.98 (t, *J* = 7.3 Hz, 2H), 1.93 - 1.75 (m, 6H); ^{13}C NMR (100 MHz, $CDCl_3$): δ 176.9, 172.6, 137.6, 136.7, 129.8, 129.7, 129.0, 128.8, 128.6, 127.0, 58.1, 51.9, 50.3, 33.9, 33.7, 30.7, 27.7, 20.6. HRMS (ESI): m/z [M + H]+ calcd for $C_{24}H_{31}N_2O_3$: 395.2329; found 395.2330. Purity was determined to be ≥95% by analytical high-performance liquid chromatography (Waters HPLC) using binary pump and Kinetex 5 µm C18

100A column (250 × 4.6 mm). UV absorption was monitored at λ = 254 nm. The injection volume was 15 μL. The gradient of acetonitrile/water (both containing 0.1% trifluoroacetic acid) was 2:98 to 90:10 over a total run time of 30 min and a flow rate of 1 mL/min. t_R = 17.4 min.

Step 5: ***Fentanyl-CRM conjugation (6)*** To a mixture of fentanyl acid **5** (0.6 mg, 2.0 equiv, 1.44 x 10^{-3} mmol), EDC (0.3 mg, 2.0 equiv, 1.44 x 10^{-3} mmol) and NHS (0.2 mg, 2.0 equiv, 1.44 x 10^{-3} mmol) in 315 μL DMSO was added. The reaction mixture was stirred at room temperature for 2 h. Then the resulting reaction mixture was added drop wise to the solution of EcoCRM (2.1 mg, 1 equiv, 7.19 x 10^{-4} mmol, dissolved in 2100 μL water) and then stirred overnight. Next, a dialysis column (Thermo Scientific) was prepared by adding approximately 44.5 mL of sterile 1X PBS into the bottom reservoir. The top reservoir was then rinsed with 500 μL of 1X PBS and placed into the bottom and reaction mixture introduced. Column was then sealed and placed onto an orbital shaker (180 rpm) for 2 hours. After agitation, the mixture was removed and placed into Eppendorf tubes and volume recorded. The mixture is then loaded into a 5 mL syringe and sterilized by filtering it through a 0.2μm HT Tuffryn membrane (Acrodisk syringe filter, Pall Corporation, Ann Arbor, MI).

References

(1) Valdez, C. A.; Leif, R. N.; Mayer, B. P. An Efficient, Optimized Synthesis of Fentanyl and Related Analogs. *PLoS One* **2014**, *9*, e108250.

(2) Bremer, P. T.; Kimishima, A.; Schlosburg, J. E.; Zhou, B.; Collins, K. C.; Janda, K. D. Combatting Synthetic Designer Opioids: A Conjugate Vaccine Ablates Lethal Doses of Fentanyl Class Drugs. *Angew. Chemie Int. Ed.* **2016**, *55*, 3772–3775.
